# Supplementary material for: Splice-Junction-Based Mapping of Alternative Isoforms in the Human Proteome
Source: Cell Rep. Author manuscript; Available in PMC 2020 Jan 15. (PMC6961840; doi:10.1016/j.celrep.2019.11.026)
Supplement: 2 [file NIHMS1546469-supplement-2.zip › DF1/PXD009737/TestisTrypsin/TestisTrypsin_36_TPM3_AISEELDHALNDMTSGSQMLKLDK.pdf]

A

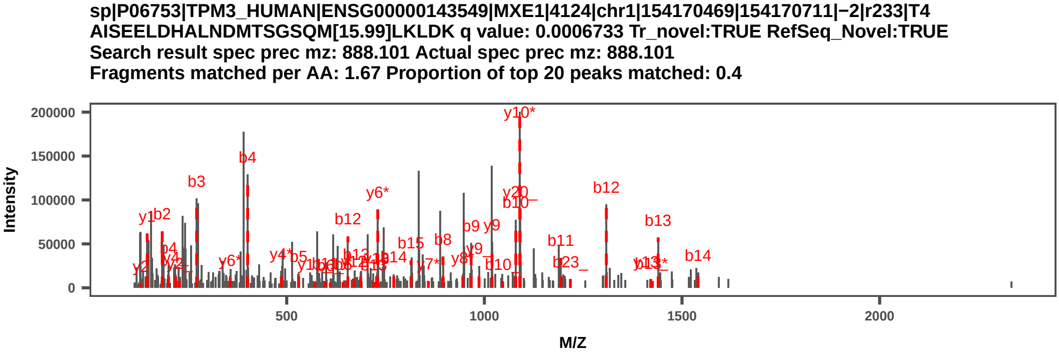
sp|P06753|TPM3_HUMAN|ENSG00000143549|MXE1|4124|chr1|154170469|154170711|−2|r233|T4
AISEELDHALNDMTSGSQM[15.99]LKLDK q value: 0.0006733 Tr_novel:TRUE RefSeq_Novel:TRUE
Search result spec prec mz: 888.101 Actual spec prec mz: 888.101
Fragments matched per AA: 1.67 Proportion of top 20 peaks matched: 0.4
Intensity
200000
150000
100000
50000
0
M/Z
500
1000
1500
2000
y10*
b4
b3
b12
y6*
b12
b13
b11
b14
b15
b8

B

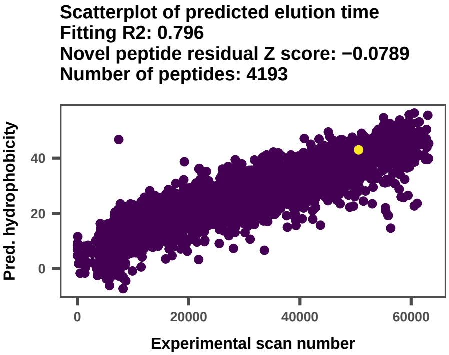
Scatterplot of predicted elution time
Fitting R2: 0.796
Novel peptide residual Z score: −0.0789
Number of peptides: 4193
Pred. hydrophobicity
40
20
0
0
20000
40000
60000
Experimental scan number

C

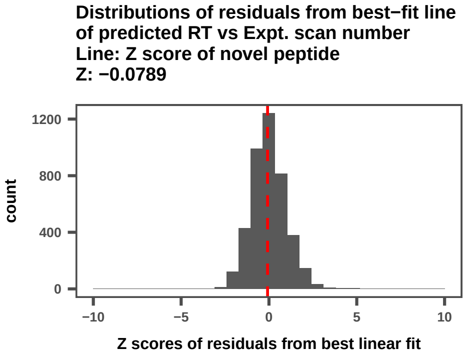
Distributions of residuals from best−fit line
of predicted RT vs Expt. scan number
Line: Z score of novel peptide
Z: −0.0789
count
1200
800
400
0
−10
−5
0
5
10
Z scores of residuals from best linear fit
